# Health Care Utilization and Clinical Characteristics of Nonhospitalized Adults in an Integrated Health Care System 28–180 Days After COVID-19 Diagnosis — Georgia, May 2020–March 2021

**DOI:** 10.15585/mmwr.mm7017e3

**Published:** 2021-04-30

**Authors:** Alfonso C. Hernandez-Romieu, Serena Leung, Armand Mbanya, Brendan R. Jackson, Jennifer R. Cope, Dena Bushman, Meredith Dixon, Jessica Brown, Tim McLeod, Sharon Saydah, Deblina Datta, Kate Koplan, Felipe Lobelo

**Affiliations:** ^1^CDC COVID-19 Response Team; ^2^Epidemic Intelligence Service, CDC; ^3^The Southeast Permanente Medical Group, Kaiser Permanente, Atlanta, Georgia; ^4^Rollins School of Public Health, Emory University, Atlanta, Georgia.

As of April 19, 2021, 21.6 million COVID-19 cases had been reported among U.S. adults, most of whom had mild or moderate disease that did not require hospitalization (*1*). Health care needs in the months after COVID-19 diagnosis among nonhospitalized adults have not been well studied. To better understand longer-term health care utilization and clinical characteristics of nonhospitalized adults after COVID-19 diagnosis, CDC and Kaiser Permanente Georgia (KPGA) analyzed electronic health record (EHR) data from health care visits in the 28–180 days after a diagnosis of COVID-19 at an integrated health care system. Among 3,171 nonhospitalized adults who had COVID-19, 69% had one or more outpatient visits during the follow-up period of 28–180-days. Compared with patients without an outpatient visit, a higher percentage of those who did have an outpatient visit were aged ≥50 years, were women, were non-Hispanic Black, and had underlying health conditions. Among adults with outpatient visits, 68% had a visit for a new primary diagnosis, and 38% had a new specialist visit. Active COVID-19 diagnoses* (10%) and symptoms potentially related to COVID-19 (3%–7%) were among the top 20 new visit diagnoses; rates of visits for these diagnoses declined from 2–24 visits per 10,000 person-days 28–59 days after COVID-19 diagnosis to 1–4 visits per 10,000 person-days 120–180 days after diagnosis. The presence of diagnoses of COVID-19 and related symptoms in the 28–180 days following acute illness suggests that some nonhospitalized adults, including those with asymptomatic or mild acute illness, likely have continued health care needs months after diagnosis. Clinicians and health systems should be aware of post-COVID conditions among patients who are not initially hospitalized for acute COVID-19 disease.

Patients aged ≥18 years who received positive results for SARS-CoV-2 (the virus that causes COVID-19) by polymerase chain reaction testing performed during April 4–September 17, 2020, and for whom ≥180 days had elapsed since their testing date were identified in KPGA EHR data. Patients were not included in the analysis if they were hospitalized in the 28 days^†^ after COVID-19 diagnosis, were pregnant during the 12 months before or at the time of COVID-19 diagnosis, or were not continuously enrolled in KPGA during the year preceding COVID-19 diagnosis.^§^ Among 3,171 patients included in the analysis, health care utilization and *International Classification of Diseases, Tenth Revision* (ICD-10) diagnostic codes were obtained for outpatient (i.e., clinic or urgent care) and emergency department visits, and hospitalizations occurring 28–180 days after COVID-19 diagnosis.^¶^

Health care utilization was determined based on the number, type (i.e., video, telephone, and in-person), setting (i.e., clinic, urgent care, emergency department, and hospital), and clinical specialty of visits. New specialty visits were defined as specialists that a patient had not consulted in the 12 months preceding COVID-19 diagnosis. New specialty visits were classified as potentially related to COVID-19 based on previously described multiorgan effects in post-COVID conditions (*2*). Clinical characteristics were ascertained through active primary and secondary** ICD-10 codes for outpatient visits. ICD-10 codes were classified as new diagnoses if they had not been documented in the 12 months preceding COVID-19 diagnosis; otherwise, they were classified as preexisting conditions.^††^ Administrative ICD-10 codes^§§^ were classified as “other.” Primary diagnoses were used to classify visit type as being for a new or preexisting condition or other. Primary and secondary diagnoses were used to describe common visits diagnoses and were classified as COVID-19–related, potentially COVID-19–related, new, or preexisting.^¶¶^ All health care utilization and clinical characteristics were described at 28–59, 60–119, and 120–180 days after COVID-19 diagnosis. Diagnoses were described as diagnosis-specific visit rates*** (visits per 10,000 person-days). Continuous variables were compared using t-tests or Wilcoxon signed-rank tests, and proportions were compared using chi-square or Fisher’s exact tests, as required. SAS (version 9.4; SAS Institute) was used to perform statistical analyses. This activity was reviewed by CDC and was conducted consistent with applicable federal law and CDC policy.^†††^

Among 3,171 identified adults with COVID-19, a total of 2,177 (69%) had one or more outpatient visits 28–180 days after COVID-19 diagnosis (Table 1). The proportion of adults with one or more visits was significantly higher among adults aged ≥65 years (88%) than among those aged 18–49 years (66%), among women (76%) than among men (59%), among non-Hispanic Black adults (71%) than among all others (68%) (p = 0.04), and among adults with three or more underlying health conditions (83%) than among those with no (60%) or one or two (69%) underlying conditions.

**TABLE 1:** Demographic and clinical characteristics of nonhospitalized COVID-19 patients with and without visits 28─180 days after initial diagnosis — Kaiser Permanente Georgia, May 19, 2020─March 16, 2021

Characteristic	Total	Outpatient visit 28─180 days after COVID-19 diagnosis			
		No. (%) of patients without visits	No. (%) of patients with ≥1 visit	Median no. of visits (IQR)*	p-value^†^
All patients	3,171	994 (31.3)	2,177 (68.7)	2 (1–5)	—
**Age group, yrs**					
18–49	**2,003**	685 (34.2)	1,318 (65.8)	2 (1–4)	<0.001
50–64	**936**	280 (29.9)	656 (70.1)	3 (1–5)	
≥65	**232**	29 (12.5)	203 (87.5)	4 (2–7)	
**Sex**					
Female	**1,796**	428 (23.8)	1,368 (76.2)	3 (1–5)	<0.001
Male	**1,375**	566 (41.2)	809 (58.8)	2 (1–4)	
**Race/Ethnicity**					
Black, non-Hispanic	**1,663**	479 (28.8)	1,184 (71.2)	3 (1–5)	<0.001
White, non-Hispanic	**870**	261 (30.0)	609 (70.0)	2 (1–5)	
Hispanic	**251**	95 (37.9)	156 (62.1)	2 (1–4)	
Asian, non-Hispanic	**126**	42 (33.3)	84 (66.7)	2 (1–4)	
**Insurance**					
Commercial	**2,724**	910 (33.4)	1,814 (66.6)	—	<0.001
Medicare	**210**	20 (9.5)	190 (90.5)	—	
**Influenza vaccination rate (%)^§^**	**1,290**	314 (24.3)	976 (75.7)	—	<0.001
**Smoker (%)^¶^**	**290**	65 (22.4)	225 (77.6)	—	0.02
Physically inactive (%)**	**906**	234 (25.8)	672 (74.2)	—	<0.001
**Underlying health conditions**					
Obesity (BMI >30)	**1,452**	391 (12.3)	1,061 (73.1)	—	<0.001
Severe obesity (BMI >40)^††^	**322**	75 (23.3)	247 (76.7)	—	0.001
Hypertension	**894**	199 (22.3)	695 (77.7)	—	<0.001
Controlled hypertension (BP <140/90 mmHg)^††, §§^	**620**	128 (20.7)	492 (79.3)	—	0.6
Diabetes	**413**	97 (23.5)	316 (76.5)	—	<0.001
Poorly controlled diabetes (HbA1c >8.0%)^††,§§^	**106**	15 (14.2)	91 (85.8)	—	0.009
Asthma	**315**	68 (21.6)	247 (78.4)	—	<0.001
Coronary artery disease	**123**	26 (21.1)	97 (78.9)	—	0.01
Congestive heart failure	**107**	19 (17.8)	88 (82.2)	—	0.002
Arrythmia	**58**	5 (8.6)	53 (91.4)	—	<0.001
Chronic obstructive pulmonary disease	**44**	5 (11.4)	39 (88.6)	—	0.004
Cancer^¶¶^	**40**	4 (10.0)	36 (90.0)	—	0.03
Chronic kidney disease***	**33**	4 (12.1)	29 (87.9)	—	0.02
End-stage renal disease***	**1**	0 (0)	1 (100)	—	0.7
HIV infection	**30**	3 (10.0)	27 (90.0)	—	0.01
**No. of underlying health conditions^†††^**					
None	**1,101**	445 (40.4)	656 (59.6)	2 (1–4)	<0.001
1 or 2	**1,405**	433 (30.8)	972 (69.2)	2 (1–4)	
3 or more	**665**	116 (17.4)	549 (82.6)	4 (2–7)	
**Charlson comorbidity index,^§§§^ mean (SD)**	**1.6 (1.3)**	1.5 (1.1)	1.7 (1.4)	—	0.02

**Abbreviations:** BMI = body mass index; HbA1c = glycated hemoglobin A1c; IQR = interquartile range; SD = standard deviation.

* Median number of visits was not calculated for some categories because of small numbers.

^†^ P-value for t-test for comparisons of means and chi-square or Fisher's exact test for categorical variables as applicable. P-value for comparisons of column percentages of patient with and without ≥1 outpatient visits.

^§^ Influenza vaccination rate during 2019–2020.

^¶^ Defined as currently smoking at the most recent clinical encounter within the last 12 months.

** Physical activity was based on patient reported weekly exercise minutes. Physical inactivity was defined as <10 minutes/week.

^††^ Represent subsets of categories in the row directly above them. Obesity, hypertension, and diabetes categories include severe obesity, controlled hypertension, and uncontrolled diabetes, respectively.

^§§^ Determined at the most recent clinical encounter within the last 12 months.

^¶¶^ Includes patients with a history of or active cancer.

*** Chronic kidney disease and end-stage renal disease are classified based on diagnosis reported by the *International Classification of Diseases, Tenth Revision* code in the patient’s medical history.

^†††^ Previous medical conditions included in this category include hypertension, diabetes, obesity, coronary artery disease, congestive heart failure, arrhythmia, asthma, chronic obstructive lung disease, chronic kidney disease, end-stage renal disease, HIV infection, or active or history of cancer.

^§§§^ Charlson comorbidity index predicts the 10-year mortality of a patient based on age and comorbidities. Scores are summed to provide a total predictive score. The lowest score of 0 corresponds to a 98% estimated 10-year survival rate. https://doi.org/10.1016/0021-9681(87)90171-8

Among adults with one or more outpatient visits, 7,991 visits occurred 28–180 days after COVID-19 diagnosis, with a median of two (interquartile range = 1–4) visits per patient (Table 2). Fewer than 2% (32) of patients were hospitalized 28–180 days after COVID-19 diagnosis. More than two thirds of patients (1,617; 68%) had visits for a new primary diagnosis. Among specialists visited, 1,627 (75%) patients visited a family, geriatric, or internal medicine provider, and 823 (38%) visited with a new specialist. Common new specialty visits potentially related to COVID-19 included dermatology (16%), behavioral/mental health (11%), gastroenterology (11%), and cardiology (10%). Overall, 58 (3%) patients saw a pulmonologist; 41 (71%) of these patients had not been evaluated by this specialty in the 12 months preceding their COVID-19 diagnosis.

**TABLE 2:** Health care visit frequency and characteristics of nonhospitalized COVID-19 patients with one or more outpatient visits 28─180 days after initial diagnosis — Kaiser Permanente Georgia, May 19, 2020─March 16, 2021

Characteristic	Total*	No. of days since COVID-19 diagnosis		
		28–59	60–119	120–180
**All patients**	**2,177**	**1,036**	**1,402**	**1,370**
No. of person-days follow-up^†^	**570,780**	98,301	187,089	190,260
**Age, yrs, median (IQR)**	**45 (32–55)**	46 (33–56)	45 (33–55)	46 (33–56)
**Visit characteristics**				
Total no. of visits	**7,991**	1,960	3,044	2,987
No. of clinic visits per patient, median (IQR)	**2 (1–4)**	1 (1–2)	2 (1–3)	2 (1–3)
**Primary visit diagnosis,^§^ no. of patients (%)**				
Preexisting diagnosis	**873 (36.7)**	341 (32.9)	494 (35.2)	432 (31.5)
New diagnosis	**1,617 (68.0)**	642 (62.0)	928 (66.2)	904 (66.0)
Other	**881 (37.0)**	267 (25.8)	402 (28.7)	397 (29.0)
**Type of visit**				
Video	**984 (45.2)**	570 (55.0)	728 (51.9)	700 (51.1)
Telephone	**1,121 (51.5)**	663 (64.0)	836 (59.6)	780 (56.9)
In-person	**1,693 (77.8)**	831 (80.2)	1,169 (83.4)	1,176 (85.8)
**Visit setting**				
Clinic^¶^	**2,177 (100.0)**	1,036 (100.0)	1,402 (100.0)	1,370 (100.0)
Urgent care	**484 (22.2)**	288 (27.8)	366 (26.1)	361 (26.4)
Emergency department	**62 (2.8)**	39 (3.8)	43 (3.1)	46 (3.4)
Hospitalization**	**32 (1.5)**	25 (2.4)	28 (2.0)	27 (2.0)
**Visit specialty**				
Primary care/Geriatrics	**1,627 (74.7)**	819 (79.1)	1,102 (78.6)	1,073 (78.3)
Behavioral health/Psychiatry	**262 (12.0)**	171 (16.5)	220 (15.7)	209 (15.3)
Dermatology	**236 (10.8)**	145 (14.0)	176 (12.6)	181 (13.2)
Cardiology	**146 (6.7)**	108 (10.4)	123 (8.8)	116 (8.5)
Gastroenterology	**134 (6.2)**	92 (8.9)	117 (8.3)	108 (7.9)
Neurology	**69 (3.2)**	52 (5.0)	60 (4.3)	57 (4.2)
Pulmonology	**58 (2.7)**	46 (4.4)	52 (3.7)	48 (3.5)
Other specialty	**1,309 (60.1)**	712 (68.7)	955 (68.1)	942 (68.8)
**New specialty visits^††,§§,¶¶^**				
Any specialist***	**823 (37.8)**	249 (24.0)	427 (30.5)	467 (34.1)
**Potentially COVID-19–related^†††^**				
Dermatology	**129 (15.7)**	31 (12.4)	52 (12.2)	59 (12.6)
Behavioral/Mental health	**92 (11.2)**	25 (10.0)	44 (10.3)	49 (10.5)
Gastroenterology	**88 (10.7)**	17 (6.8)	48 (11.2)	38 (8.1)
Cardiology	**79 (9.6)**	34 (13.7)	35 (8.2)	33 (7.1)
Otolaryngology	**63 (7.7)**	14 (5.6)	34 (8.0)	33 (7.1)
Pulmonology	**41 (5.0)**	13 (5.2)	19 (4.4)	16 (3.4)
Neurology	**32 (3.9)**	8 (3.2)	13 (3.0)	18 (3.9)
**Other**				
Gynecology	**167 (20.0)**	43 (17.3)	87 (20.4)	76 (16.3)
Orthopedic surgery	**101 (12.3)**	22 (8.8)	41 (9.6)	58 (12.4)
Ophthalmology	**56 (6.8)**	16 (6.4)	14 (3.3)	30 (6.4)
General surgery	**51 (6.2)**	11 (4.4)	27 (6.3)	29 (6.2)
Urology	**36 (4.4)**	9 (3.6)	18 (4.3)	18 (3.9)

**Abbreviations:** EHR = electronic health record; ICD-10 = *International Classification of Diseases, Tenth Revision;* IQR = interquartile range.

* Frequencies in this column do not represent the sum of time-interval specific frequencies because some patients might have had more than one visit within or across periods.

^†^ Person-time was calculated for all patients with possible follow-up 28–180 days after COVID-19 diagnosis (n = 3,171).

^§^ Primary visit diagnosis was classified as preexisting diagnosis if the primary diagnosis for a visit had been recorded in a patient's EHR in the 12 months before COVID-19 diagnosis, new diagnosis if the primary diagnosis was not recorded in a patient's EHR in the 12 months before COVID-19 diagnosis, and other if the primary diagnosis was a screening or administrative code (i.e., ICD-10 codes Z00–Z99). ICD-10 codes Z00–Z99 are factors influencing health status and contact with health services and include encounters for general adult medical examination, encounters for administrative examination (e.g., preemployment and insurance purposes), and encounters for screening for disease (e.g., infectious and parasitic diseases and malignant neoplasms), among others.

^¶^ Clinic visits include in-person, video, and telephone visits.

** Hospitalizations for any diagnosis that occurred 28–180 days after initial COVID-19 diagnosis.

^††^ New specialist visits were defined as any visit that occurred after SARS-CoV-2 infection diagnosis in a specialty in which a patient had not been seen in the 12 months before their COVID-19 diagnosis. New specialty visit numbers are a subset of the total outpatient visit specialty category. For example, 41 (71%) of 58 patients who had a pulmonology visit were new patients in this specialty.

^§§^ Proportions of new specialist visits use the total of patients with a new specialist visit (n = 823) as the denominator rather than all adults with outpatient visits (n = 2,177).

^¶¶^ The order of the 12 most common new specialist visits were as follows: 1) gynecology, 2) dermatology, 3) orthopedic surgery, 4) behavioral/mental health, 5) gastroenterology, 6) cardiology, 7) otolaryngology, 8) ophthalmology, 9) general surgery, 10) pulmonology, 11) urology, and 12) neurology.

*** Excludes primary care/geriatrics, adult urgent care, podiatry, and optometry.

^†††^ Specialties potentially related to COVID-19 were based on descriptions of symptoms and multiorgan effects in post-COVID conditions. https://doi.org/10.1038/s41591-021-01283-z

COVID-19 was recorded as an active diagnosis for 210 (10%) of 2,177 patients who had one or more outpatient visit within 180 days of COVID-19 diagnosis (Table 3). COVID-19–related visits declined from 24 per 10,000 person-days during the 28–59-day interval to fewer than two per 10,000 person-days during the 120–180-day interval. Visits per 10,000 person-days for symptoms potentially related to COVID-19 declined during these same intervals, including those for throat or chest pain (from seven per 10,000 person-days to four), shortness of breath (from eight to three), cough (from four to two), and malaise and fatigue (from four to two). In contrast, rates of visits with chronic disease diagnoses (e.g., hypertension and diabetes) and urinary tract infections changed little over time.

**TABLE 3:** Twenty most common new and preexisting outpatient diagnoses among nonhospitalized COVID-19 patients with one or more outpatient visits 28─180 days after initial diagnosis — Kaiser Permanente Georgia, May 19, 2020─March 16, 2021

Characteristic	Total	No. of days since COVID-19 diagnosis		
		28–59	60–119	120–180
**All patients**	**2,177**	**1,036**	**1,402**	**1,370**
No. of person-days follow-up*	**570,780**	98,301	187,089	190,260
**Diagnoses^†,§,¶^ (ICD-10 code)**	**No. (%) of patients**	**No. of visits (visits per 10,000 person-days)**		
COVID-19 (U07, J12, J20, B94)**	**210 (9.6)**	235 (23.9)	62 (3.3)	29 (1.5)
**Symptoms potentially related to COVID-19^††^**				
Pain in throat and chest (R07)	**145 (6.7)**	69 (7.0)	79 (4.2)	68 (3.6)
Shortness of breath/dyspnea (R06)	**128 (5.9)**	78 (7.9)	59 (3.2)	54 (2.8)
Headache (R51)	**101 (4.6)**	37 (3.8)	53 (2.8)	48 (2.5)
Malaise and fatigue (R53)	**96 (4.4)**	35 (3.6)	43 (2.3)	40 (2.1)
Cough (R05)	**86 (4.0)**	41 (4.2)	33 (1.8)	30 (1.6)
Sleep disorders (G47)	**80 (3.7)**	24 (2.4)	41 (2.2)	44 (2.3)
Abnormalities of heartbeat^§§^ (R00)	**68 (3.1)**	42 (4.3)	37 (2.0)	25 (1.3)
**New diagnoses**				
Back pain (M54)	**219 (10.1)**	80 (8.1)	94 (5.0)	135 (7.1)
Joint disorder (M25)	**211 (9.7)**	50 (5.1)	113 (6.0)	133 (7.0)
Muscle or soft tissue disorder (M79)	**172 (7.9)**	53 (5.4)	92 (4.9)	100 (5.3)
Abdominal and pelvic pain (R10)	**167 (7.7)**	52 (5.3)	100 (5.3)	84 (4.4)
Anxiety (F41)	**96 (4.4)**	33 (3.4)	51 (2.7)	68 (3.6)
Hyperlipidemia (E78)	**96 (4.4)**	25 (2.5)	42 (2.2)	43 (2.3)
Overweight/Obesity (E66)	**96 (4.4)**	23 (2.3)	43 (2.3)	42 (2.2)
Urinary tract infection and urinary incontinence (N39)	**76 (3.5)**	14 (1.4)	34 (1.8)	38 (2.0)
Hypertension (I10)	**73 (3.4)**	27 (2.7)	48 (2.6)	33 (1.7)
Diabetes mellitus (E11)	**72 (3.3)**	24 (2.4)	39 (2.1)	49 (2.6)
Disorders of refraction and accommodation (H52)	**67 (3.1)**	12 (1.2)	29 (1.6)	31 (1.6)
Gastresophageal reflux (K21)	**67 (3.1)**	19 (1.9)	29 (1.6)	38 (2.0)
**Preexisting diagnoses**				
Hypertension (I10)	**343 (15.8)**	127 (12.9)	216 (11.5)	201 (10.6)
Diabetes (E11)	**211 (9.7)**	96 (9.8)	168 (9.0)	143 (7.5)
Overweight/Obesity (E66)	**123 (5.6)**	28 (2.8)	61 (3.3)	57 (3.0)
Hyperlipidemia (E78)	**120 (5.5)**	34 (3.5)	60 (3.2)	54 (2.8)
Anxiety (F41)	**89 (4.1)**	48 (4.9)	86 (4.6)	63 (3.3)
Back pain (M54)	**63 (2.9)**	21 (2.1)	43 (2.3)	40 (2.1)
Cough (R05)	**63 (2.9)**	39 (4.0)	33 (1.8)	29 (1.5)
Major depressive disorder (F33)	**50 (2.3)**	34 (3.5)	58 (3.1)	38 (2.0)
Asthma (J45)	**49 (2.3)**	22 (2.2)	21 (1.1)	31 (1.6)
Attention-deficit hyperactivity disorders (F90)	**43 (2.0)**	16 (1.6)	38 (2.0)	29 (1.5)
Sleep disorders (G47)	**41 (1.9)**	14 (1.4)	25 (1.3)	22 (1.2)
Pain in throat and chest (R07)	**38 (1.7)**	21 (2.1)	17 (0.9)	18 (0.9)
Joint disorder (M25)	**37 (1.7)**	7 (0.7)	18 (1.0)	18 (0.9)
Gastroesophageal reflux (K21)	**35 (1.6)**	11 (1.1)	19 (1.0)	17 (0.9)
Shortness of breath/dyspnea (R06)	**35 (1.6)**	30 (3.1)	9 (0.5)	13 (0.7)
Abdominal and pelvic pain (R10)	**34 (1.6)**	7 (0.7)	27 (1.4)	12 (0.6)
Chronic ischemic heart disease (I25)	**34 (1.6)**	12 (1.2)	26 (1.4)	20 (1.1)
Glaucoma (H40)	**33 (1.5)**	12 (1.2)	22 (1.2)	21 (1.1)
Hypothyroidism (E03)	**33 (1.5)**	4 (0.4)	17 (0.9)	20 (1.1)
Chronic kidney disease (N18)	**29 (1.3)**	15 (1.5)	14 (0.7)	11 (0.6)

**Abbreviation:** ICD-10 = *International Classification of Diseases, Tenth Revision*.

* Person-time was calculated for all patients with possible follow-up 28–180 days after COVID-19 diagnosis (n = 3,171).

^†^ A diagnosis was considered active if providers billed for it during a visit under the assumption it coexisted at the time of the visit and required or affected patient care, treatment, or management. “History of” diagnostic codes were not included in descriptions. For example, patients with a history of COVID-19 diagnosis that was not considered active were not included.

^§^ New diagnoses were defined as three-letter ICD-10 diagnosis codes that were not recorded in a patient’s electronic health record in the 12 months before SARS-CoV-2 infection diagnosis.

^¶^ Preexisting diagnoses were defined as three-letter ICD-10 diagnosis codes that were recorded in a patient’s electronic health record in the 12 months before SARS-CoV-2 infection diagnosis.

** U07 includes only U07.1; J12 and J20 were included if the associated written diagnosis included COVID-19, SARS-CoV-2, or coronavirus. Approximately 95% of diagnoses in this category were U07.1.

^††^ This list is not exhaustive and is based on ongoing or new symptoms ≥4 weeks after COVID-19 diagnosis commonly reported in the scientific literature.

^§§^ Includes palpitations, tachycardia, and bradycardia.

## Discussion

Among adult patients with COVID-19 and enrolled in an integrated health system in Georgia, who were not hospitalized for their acute illness, approximately two thirds had at least one outpatient medical encounter 28–180 days after diagnosis, and approximately two thirds of these persons received a new primary diagnosis at one or more visits. New diagnoses included cough, shortness of breath, chest or throat pain, and fatigue, which likely represent ongoing COVID-19 symptoms and are consistent with other reports of patient-reported symptoms months after SARS-CoV-2 infection (*3*–*7*). Although the frequency of visits for these symptom diagnoses decreased after 60 days, they persisted beyond 120 days among some patients. Clinicians and health care systems should be aware of the possibility of medical encounters related to a previous diagnosis of COVID-19 beyond the acute illness.

Whether the number of visits among nonhospitalized adults 28–180 days after COVID-19 diagnosis is higher compared with adults without COVID-19 remains unclear. However, compared with health care utilization among nonhospitalized adults with influenza in Spain during the 2009 H1N1 pandemic, the mean number of outpatient visits in the 120 days after diagnosis was higher in adults with COVID-19 (mean = 2.2, standard deviation = 1.7 among adults with positive test results for SARS-CoV-2; mean = 1.7, standard deviation = 1.3 among adults with positive test results for H1N1) (*8*). 

More than one in three (38%) patients underwent a new specialist evaluation. Some of the common new specialist visits (e.g., gynecology, orthopedic and general surgery, and urology) are likely unrelated to COVID-19 and might have occurred because of specialty visits missed or avoided during stay-at-home orders or local increases in COVID-19 cases. Specialties related to multiorgan effects of post-COVID conditions (*2*), such as pulmonology, neurology, cardiology, and behavioral/mental health, were common in this patient population, indicating that nonhospitalized adults might be referred for additional evaluation for COVID-19–related symptoms and conditions after the acute illness.

The prevalence of underlying health conditions was higher among patients with outpatient visits ≥28 days after the initial COVID-19 diagnosis than among those without such visits; this finding might be explained by increased engagement in care among patients with chronic medical problems. Some underlying health conditions in the study population, such as obesity and diabetes, are associated with increased risk for hospitalization and death from COVID-19 (*9*); however, whether underlying health conditions increase the risk for post–COVID-19 conditions remains unclear.

A higher proportion of women and non-Hispanic Black adults had one or more outpatient visits than did men and adults of other racial or ethnic groups. This could be a result of certain groups being disproportionately affected by COVID-19 (*9*), differences in care seeking and in the prevalence of underlying health conditions, or a higher risk for post–COVID-19 conditions among these populations. Women might be at higher risk for persistent pulmonary dysfunction and symptoms after SARS-CoV-2 infection (*10*); however, studies of post–COVID-19 conditions have not reported race/ethnicity (*3*–*5*) or have had low representation by certain racial or ethnic groups (*7*). Future evaluations of post–COVID-19 conditions should include diverse racial/ethnic groups and examine differences by sex and race/ethnicity to guide health care planning and estimates of health care utilization.

The findings in this report are subject to at least six limitations. First, approximately three quarters of adults included in this study were commercially insured patients in a single integrated health system whose health care utilization might differ from that of other U.S. populations, including uninsured or publicly insured adults. Examining records in other health systems is needed to confirm these findings. Second, use of diagnostic symptom codes by providers might not record all symptoms. Third, without a non–COVID-19 control group, it was not possible to evaluate associations between COVID-19 and diagnostic codes and health care utilization. Fourth, 12-month retrospective reviews of diagnostic codes and specialty visits might have missed previous diagnoses and care. Fifth, it is unclear whether the use of a COVID-19 diagnosis visit code was used by providers for patients with prolonged symptoms or clinical findings from the initial SARS-CoV-2 infection. Finally, it was not possible to determine whether patients might have been experiencing symptoms of reinfection with SARS-CoV-2, rather than ongoing COVID-19 symptoms.

Approximately two thirds of nonhospitalized patients sought medical care 28–180 days after their COVID-19 diagnosis. The presence of active COVID-19, symptoms of COVID-19 diagnoses, and specialty referrals suggest that some nonhospitalized adults, including those with asymptomatic or mild acute illness, likely have continued health care needs months after diagnosis. Raising awareness among patients, clinicians, and health systems about common new diagnoses and health needs, including specialist evaluation, after acute SARS-CoV-2 infection is important to understand the long-term effects of the illness.

**Summary**

**What is already known about this topic?**

Health care needs in the months after a COVID-19 diagnosis among nonhospitalized adults have not been well studied.

**What is added by this report?**

Among 3,171 nonhospitalized adult COVID-19 patients, 69% had one or more outpatient visits 28–180 days after the diagnosis. Two thirds had a visit for a new primary diagnosis, and approximately one third had a new specialist visit. Symptoms potentially related to COVID-19 were common new visit diagnoses. Visits for these symptoms decreased after 60 days but for some patients continued through 120–180 days.

**What are the implications for public health practice?**

Clinicians and health care systems should be aware of the potential for post-COVID conditions.

## Footnotes

* A diagnosis was considered active if providers billed for it during a visit under the assumption it coexisted at the time of the visit and required or affected patient care, treatment, or management. “History of” diagnostic codes were not included in descriptions. For example, patients with a history of COVID-19 diagnosis that was not considered active were not included. ^†^ Restriction of the analytic sample to patients not hospitalized within 28 days of COVID-19 diagnosis and seeking care ≥28 days after diagnosis was used to exclude adults with severe COVID-19 and adults with mild or moderate disease still in the acute phase of infection.

§ A total of 4,646 laboratory-confirmed COVID-19 cases occurred during April 4–September 17, 2020. The final analytic sample was 3,171 patients after exclusion of patients hospitalized in the first 28 days after SARS-CoV-2 diagnosis (281), patients aged <18 years (337), patients without 12 months of continuous enrollment in KPGA before their COVID-19 diagnosis (783), patients with Medicaid (one), and patients pregnant in the 12 months before or at the time of COVID-19 diagnosis (73).

¶ The follow-up period for the total cohort of patients was May 19, 2020–March 16, 2021.

** Primary visit diagnosis refers to the first-listed diagnosis after an outpatient visit that indicates the diagnosis chiefly responsible for a patient’s visit. Secondary diagnoses include all other diagnoses listed at a visit after the primary diagnosis.

†† A 12-month retrospective EHR review was performed to determine whether ICD-10 codes were preexisting or new. Retrospective review of ICD-10 codes was performed at the three-letter level (e.g., I10, F22, and R00).

§§ Administrative ICD-10 codes include codes Z00–Z99 and R70–R99.

¶¶ Descriptions of common diagnoses excluded administrative ICD-10 codes. Diagnoses potentially related to COVID-19 included symptom diagnoses such as cough, shortness of breath, and fatigue. Diagnoses potentially related to COVID-19 were only counted if they had not been documented in a patient’s EHR in the 12 months preceding COVID-19 diagnosis.

*** Person-time used to calculate rates of visits for a given diagnosis were calculated by determining the time from first SARS-CoV-2 positive test collection date to March 16, 2021. Once the total follow-up time was determined per patient, each patients’ contribution to intervals of 28–59, 60–119, and 120–180-days was determined. Data for patients with >180 days of follow-up was truncated so that all outpatient visits occurring after 180 days were excluded from analysis.

††† 45 C.F.R. part 46, 21 C.F.R. part 56; 42 U.S.C. Sect. 241(d); 5 U.S.C. Sect. 552a; 44 U.S.C. Sect. 3501 et seq.
